# Cost‐Effectiveness Analysis of Nirsevimab for Respiratory Syncytial Virus Disease Prevention in Newborns of Hong Kong

**DOI:** 10.1111/irv.70153

**Published:** 2025-10-01

**Authors:** Yingcheng Wang, Mingjun Rui, Qiran Wei, Ting Fan Leung, Joyce H. S. You

**Affiliations:** ^1^ School of Pharmacy, Faculty of Medicine The Chinese University of Hong Kong Hong Kong SAR China; ^2^ Department of Paediatrics, Faculty of Medicine The Chinese University of Hong Kong Hong Kong SAR China

**Keywords:** catch‐up schedule, cost‐effectiveness, newborns, nirsevimab, seasonal immunization schedule, year‐round immunization schedule

## Abstract

**Background:**

Nirsevimab, a long‐acting monoclonal antibody, was shown to prevent respiratory syncytial virus (RSV) infections in newborns. We evaluated the cost‐effectiveness of nirsevimab strategies for newborns from the societal perspective in Hong Kong.

**Methods:**

A Markov model was developed to simulate outcomes of four nirsevimab strategies in newborns: (1) year‐round, (2) seasonal, (3) catch‐up, and (4) no nirsevimab. Primary outcomes included RSV lower respiratory tract infections (LRTI) related events, direct and indirect costs, quality‐adjusted life year (QALY) loss, and incremental cost per QALY (ICER).

**Results:**

In base‐case analysis, all strategies with nirsevimab reduced RSV–LRTI‐related events. The catch‐up group had the lowest QALY loss per 100,000 infants (38.82), followed by year‐round (45.71), seasonal (60.60), and no intervention groups (81.52). Three nirsevimab cost levels were examined: 10%, 25%, and 50% of the US cost. At 10% US cost (USD52), all strategies were cost‐saving versus no intervention. At 25% US cost (USD130), the ICER of the catch‐up group (vs. no intervention) was 141,925 USD/QALY. At 50% US cost (USD260), all nirsevimab strategies were not cost‐effective versus no intervention at a willingness‐to‐pay of 162,401 USD/QALY. Influential factors with thresholds were identified for RSV‐LRTI incidence, RSV‐related hospitalization and mortality, and nirsevimab effectiveness at the 25% US cost level (USD130). In probabilistic sensitivity analysis, the catch‐up and no intervention strategies were cost‐effective 100% of the time at 10% (USD52) and 50% (USD260) US cost, respectively. At 25% US cost (USD130), the catch‐up strategy was cost‐effective 58.56% of the time.

**Conclusions:**

The cost‐effectiveness acceptance of nirsevimab was highly subject to drug cost and effectiveness of nirsevimab, RSV‐LRTI incidence, and RSV–LRTI‐related consequences.

## 1 Introduction

Respiratory syncytial virus (RSV) is the leading cause of lower respiratory tract infections (LRTI) and a major contributor to infant hospital admissions [1, 2, 3]. Globally, it is estimated that RSV is responsible for 33 million LRTI episodes, 3.6 million hospital admissions, 26,300 hospital‐related deaths, and 101,400 total deaths annually in children under 5 years old [4]. The morbidity associated with RSV infection in infants incurs a considerable burden to the Hong Kong healthcare system. A 15‐year retrospective study in Hong Kong reported that RSV ranked top among causes of hospitalization in children aged under 5 years, with an annual hospitalization incidence of 139.3–175.1 per 10,000 population and a mortality of 12.8–22.4 per 10,000 hospitalizations [5].

Nirsevimab, a long‐acting monoclonal antibody that prevents viral entry into cells by targeting the RSV F protein [6], has been approved for single‐dose administration to infants in Europe, Canada, and the United States for the prevention of RSV‐related LRTI. Two clinical trials (*n* = 2350) have demonstrated significant efficacy of nirsevimab in both preterm and term infants [7, 8], with a pooled relative risk reduction of 79.5% (95% CI: 65.9%–87.7%) for medically attended RSV LRTI when comparing with the placebo group [9].

Despite the promising clinical outcomes of the long‐acting monoclonal antibody for RSV prevention in infants, the cost‐effectiveness of nirsevimab in Hong Kong is yet to be investigated. To inform decision‐makers on policy development and resource allocation for RSV prevention in infants, this study aimed to evaluate the potential cost‐effectiveness of various nirsevimab immunization strategies from a societal perspective in Hong Kong.

## 2 Method

### 2.1 Model Design

**FIGURE 1:**
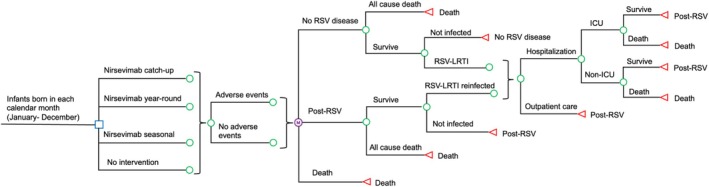
Simplified Markov model. ICU, intensive care unit; LRTI, lower respiratory tract infections; RSV, respiratory syncytial virus.

A Markov model (Figure 1) was designed to simulate the RSV LRTI‐related economic and clinical outcomes in a hypothetical cohort of newborns who were born over 12 calendar months. The Markov model is a type of decision‐analytical model in which the hypothetical subjects transit between health states in subsequent cycles to simulate the cumulative costs and healthcare outcomes over time [10]. Four immunization strategies were examined: (1) nirsevimab administered at birth to newborns who were born throughout the year (nirsevimab year‐round group); (2) nirsevimab administered at birth to newborns who were born during the RSV season (nirsevimab seasonal group); (3) nirsevimab administered at birth to newborns who were born during the RSV season and to infants who were born outside the season at the onset of RSV season (nirsevimab catch‐up group); and (4) no nirsevimab was administered (no intervention group). The model applied March to September as the RSV season in Hong Kong, based upon RSV sentinel surveillance data collected locally between 1998 and 2015 [11]. The time horizon of the Markov model was 1 year with a monthly cycle. Primary outcomes measured included direct medical costs, indirect costs, incidence of RSV‐LRTI infection, RSV–LRTI‐related hospitalization, intensive care unit (ICU) admission, death, and quality‐adjusted life‐years (QALY) loss. The completed CHEERS checklist [12] of this study is shown in Data S1.

The hypothetical newborns entered the model at the “no RSV” Markov health state. In the three nirsevimab groups, the newborns might or might not experience adverse events of grade ≥ 3 severity (gastroenteritis, bronchiolitis, and otitis media) following immunization. All newborns, with or without nirsevimab immunization, were at risk of all‐cause mortality in each monthly cycle. Infants who survived in each monthly cycle might acquire RSV‐LRTI and receive treatment in outpatient or inpatient settings. Infants who were hospitalized for RSV‐LRTI might or might not survive (with or without ICU admission). Infants who had recovered from RSV‐LRTI transited to “post‐RSV” Markov state and were at risk of all‐cause death and RSV‐LRTI reinfection in the subsequent cycle.

### 2.2 Clinical Inputs

A literature search was conducted in MEDLINE from 2000 to 2024 using keywords such as “RSV infection,” “nirsevimab,” “infant,” “medical utilization,” “hospitalization,” and “mortality.” The inclusion criteria were (1) research articles in English; (2) study subjects were infants (1 year of age or younger and were entering their first RSV season); and (3) nirsevimab‐related adverse events or RSV‐related events were reported. Meta‐analyses and randomized controlled trials were preferred sources of clinical inputs, and case reports were excluded. In addition to data reported by clinical trials, the sensitivity analysis ranges also included the findings reported in real‐world effectiveness publications. All model inputs are listed in Table 1.

**TABLE 1:** Model inputs.

Parameters	Base‐case value	Range for sensitivity analysis	Distribution	Reference
**Clinical inputs**				
Newborn distribution			Dirichlet	[13]
January	0.087	0.076–0.102		
February	0.077	0.066–0.087		
March	0.083	0.076–0.090		
April	0.074	0.065–0.081		
May	0.080	0.072–0.086		
June	0.078	0.072–0.090		
July	0.081	0.070–0.090		
August	0.084	0.073–0.089		
September	0.083	0.079–0.087		
October	0.091	0.084–0.103		
November	0.092	0.080–0.107		
December	0.090	0.079–0.104		
Yearly RSV‐LRTI incidence rate in infants	0.0379	0.0318–0.044	Beta	[4, 11, 14]
Relative RSV‐LRTI incidence ratio of in‐season versus off‐season	3.01	2.41–3.61	Beta	[4, 11, 14]
RSV‐LRTI hospitalization rate among RSV infected infants	0.571	0.457–0.685	Beta	[4]
Proportion of ICU admission among RSV–LRTI related hospitalization	0.024	0.019–0.029	Beta	[15]
Mortality rate of RSV–LRTI related hospitalization	0.00042	0.00034–0.00051	Beta	[5, 16]
Monthly probability of all‐cause mortality for infants	0.00014	0.00011–0.00017	Beta	[17]
RSV‐LRTI monthly reinfection rate	0.00053	0.00043–0.00064	Beta	[18]
**Nirsevimab effectiveness against**				
RSV‐LRTI at 5 months	0.795	0.659–0.877	Beta	[9, 19]
Hospitalization at 5 months	0.773	0.503–0.897	Beta	[9, 19]
ICU admission at 5 months	0.860	0.625–0.948	Beta	[9, 19]
Incidence rate of nirsevimab‐associated adverse events ≥ grade 3^a^	0.036	0.029–0.043	Beta	[8]
Nirsevimab coverage	100%	—	Uniform	Assumption
**Utility inputs**				
Disutility score of adverse events ≥ grade 3	0.098	0.006–0.19	Beta	[20]
Disutility score of RSV‐LRTI outpatients	0.16	0.121–0.199	Beta	[21]
Disutility score of RSV‐LRTI hospitalization	0.41	0.351–0.469	Beta	[21]
Disutility score of RSV‐LRTI ICU admission	0.6	0.541–0.659	Beta	[21]
Length of hospitalization (non‐ICU) (days)	3	1.3–9.01	Triangular	[19, 22]
Length of ICU (survival) (days)	5	4–10.3	Triangular	[15]
Length of ICU (death) (days)	19.5	8.8–33	Triangular	[15]
**Cost inputs**				
Nirsevimab price in the US (USD per dose)	519.75	—	—	[23]
Cost of outpatient care (USD per clinic visit)	57	46–68	Gamma	[24]
Cost of hospitalization (USD per day)	654	523–785	Gamma	[24]
Cost of ICU (USD per day)	3128	2503‐3754	Gamma	[24]
Number of clinic visits for RSV outpatient care	2	1–3	Triangular	Assumption
Number of clinic visits for adverse events ≥ grade 3	1	1–2	Triangular	Assumption
Length of illness for RSV infection (days)	14	11.2–16.8	Triangular	[25]
Labor force participation rate	0.831	0.795–0.865	Beta	[26]
Unemployment rate	0.034	0.025–0.074	Beta	[26]
Median monthly earning of employed persons (USD)	2539	2031–3046	Gamma	[27]

Abbreviations: ICU, intensive care unit; LRTI, lower respiratory tract infections; RSV, respiratory syncytial virus.

^a^ The adverse events of grade ≥ 3 severity after nirsevimab infection that required medical care (such as gastroenteritis, bronchiolitis, and otitis media) were included in the model.

The distributions of births over 12 calendar months were based on the birth numbers in each calendar month in 2024 reported by the Census and Statistics Department of Hong Kong [13]. The RSV disease burden in children aged 0–12 months in high‐income regions (RSV‐related LRTI incidence rate 38.5 per 1000 children per year, and RSV‐related LRTI hospital admission rate 22.0 per 1000 children per year) [4] was used to estimate the hospitalization rate among infected infants (22/38.5 = 57.1%). The yearly RSV‐LRTI incidence rate in infants (3.79%) and relative RSV‐LRTI incidence ratio of in‐season versus off‐season (3.01) were estimated from the RSV hospitalizations in infants (reported by a cost‐effective study of monoclonal antibody using the Hong Kong RSV sentinel surveillance data in 1998–2015 [11]), the infant population in 1998–2015 [14], and hospitalization rate among infected infants [4]. Details of estimates are shown in Table S1. The proportion of ICU admission among RSV–LRTI‐related hospitalization (2.4%) was retrieved from a retrospective study (*n* = 4912 RSV‐positive cases in 2009–2011) on RSV–LRTI‐associated pediatric ICU admissions in Hong Kong [15]. The mortality rate of hospitalized RSV‐infected infants was obtained from a systematic review and meta‐analysis of 44 studies (149,321,171 participants), which estimated the RSV‐related disease burden in children aged 5 years and younger [16]. All‐cause mortality of infants was adopted from Hong Kong life table [17]. The monthly RSV‐LRTI reinfection rate was estimated from findings of a multicenter retrospective case–control study (*n* = 3538) of hospitalized children with RSV infections during 2013–2015 in Hong Kong [18].

RSV‐LRTI event rate in the immunized groups was estimated using the following formula: RSV‐LRTI event rate*(1 − nirsevimab effectiveness against the event). The effectiveness of nirsevimab against RSV‐LRTI, hospitalization, and ICU admission was derived from a pooled analysis (*n* = 2350) [9] of two randomized controlled trials of nirsevimab in preterm infants and healthy late‐preterm [7, 8]. Nirsevimab was estimated to reduce RSV‐LRTI incidence by 79.5% (95% CI: 65.9%–87.7%) for up to 5 months post‐dose. The estimated effectiveness against hospitalization and very severe RSV‐LRTI was 77.3% (95% CI: 50.3%–89.7%) and 86% (95% CI: 62.5%–94.8%), respectively. A meta‐analysis of 27 observational studies [19] showed the real‐world effectiveness of nirsevimab against RSV‐LRTI and hospitalization was within the 95% CI ranges reported by the pooled analysis of clinical trials [9]. The effectiveness against ICU admission of the model adopted the reported effectiveness against very severe RSV‐LRTI. As no evidence of nirsevimab effectiveness against death was reported in clinical trials or real‐world studies, the RSV‐related mortality rate was applied for all groups. To account for effectiveness beyond 5 months, the present model applied a linear decline (adopted from previous studies [21, 28]), reaching zero effectiveness at month 10 post‐dose. The incidence rate (0.036) of adverse events ≥ 3 grade following nirsevimab injection was also retrieved from the clinical trials [7, 8]. The coverage of nirsevimab in all nirsevimab strategies was assumed to be 100%.

### 2.3 Utility Inputs

The RSV‐related QALY loss over a one‐year time horizon was estimated using the time spent in each RSV‐related health event and the associated utility loss for each event. The RSV‐related events included: (1) adverse events of nirsevimab, (2) outpatient care for RSV‐LRTI, (3) hospitalization for RSV‐LRTI, (4) ICU admission, and (5) RSV‐associated death. The disutility value for adverse events ≥ 3 grade following nirsevimab injection was derived from a study that estimated health state utilities associated with pneumonia and acute otitis media in children 0–5 years using trade‐off approaches [20]. Disutility values for RSV‐LRTI related outpatient care, hospitalization, and ICU admission were drawn from a cost‐effectiveness analysis on the prevention of infant RSV infection [21]. The length of non‐ICU hospital stay was obtained from a three‐year retrospective study of 670 children with laboratory‐confirmed RSV infections in Hong Kong [22] and a meta‐analysis (including 27 observational studies) of the real‐world effectiveness of nirsevimab in infants [19]. The duration of ICU stay (survival and deaths) was retrieved from a 30‐month retrospective epidemiology study on RSV‐associated pediatric ICU admissions in Hong Kong (*n* = 4912) [15]. The QALY loss of RSV‐LRTI related death was calculated using the remaining life expectancy derived from the Hong Kong life table and age‐specific utility values [17, 29]. The QALY loss was discounted with an annual discount rate of 3%.

### 2.4 Costs Inputs

The economic analysis was conducted from a societal perspective of Hong Kong, accounting for both direct medical costs and indirect costs. Direct costs included costs of nirsevimab administration, treatment of adverse events, and outpatient and inpatient care for RSV‐LRTI from the perspective of a public healthcare provider in Hong Kong. Nirsevimab is available in Hong Kong, but not yet included in the drug formulary of the public healthcare provider. To explore the cost‐effectiveness at various nirsevimab cost levels, the present analysis therefore benchmarked US pricing (USD519.75 per dose [23]) at three cost levels: 10% (USD52), 25% (USD130), and 50% (USD260) in three sets of base‐case analysis.

The unit costs of general outpatient clinic visits, hospital days (for general medical wards) and ICU days were estimated using the Hospital Authority 2024 fee schedule [24]. In the base‐case analysis, it was assumed that each RSV‐LRTI outpatient care included two clinic visits (ranged 1–3 visits for sensitivity analysis). Management of adverse events of nirsevimab was assumed to require at least one outpatient visit (range 1–2 visits). Indirect costs included productivity loss of caregiver time and premature death due to RSV‐associated mortality. Productivity loss estimates were based on age‐specific labor force participation rates, employment rates, median monthly income [26, 27], and the average duration of illness‐related work absences for caregivers [25] and productivity loss over age 18–65 years in premature deaths. Cost was discounted to the current year with an annual discount rate of 3%.

### 2.5 Cost‐Effectiveness Analysis, Sensitivity Analysis, and Scenario Analysis

All analyses were performed using TreeAge Pro 2024 (TreeAge Software Inc., Williamstown, MA, USA) and Microsoft Excel 365 (Microsoft Corporation, Redmond, WA, USA). In the cost‐effectiveness analysis, a strategy was considered dominated and thus excluded if it resulted in a net QALY loss at a higher cost when compared to an alternative option. A strategy was considered cost‐effective if it resulted in (1) net QALY gain with a cost‐saving (dominant) or (2) net QALY gain with an incremental cost and the incremental cost‐effectiveness ratio (ICER = Incremental cost/QALY gain) was lower than the willingness‐to‐pay (WTP) threshold. According to the World Health Organization, an ICER below 1 × GDP per capita is considered highly cost‐effective, while an ICER below 3 × GDP per capita is considered cost‐effective [30]. The GDP per capita in Hong Kong was USD54,134 in 2024 [31], and the present model adopted 3 × GDP per capita (162,401 USD/QALY) as the WTP threshold.

One‐way sensitivity analyses were conducted on all model inputs, using the 95% CI or range (or ±20% if 95% CI and range were not available) of the base‐case value. A drug cost threshold analysis was performed by extending the lower limit of nirsevimab cost to USD 0. To assess uncertainties across all variables, a probabilistic sensitivity analysis using Monte Carlo simulation was conducted. The total cost and QALY loss for all four groups were recalculated 10,000 times by randomly drawing all model inputs simultaneously from the variable‐specific distributions listed in Table 1. The probability of each immunization strategy to be the preferred option was evaluated across a range of WTP thresholds and presented in the cost‐effectiveness acceptability curves.

Scenario analyses were conducted to explore the cost‐effectiveness of nirsevimab in the following scenarios: Scenario 1 modeled an additional strategy of palivizumab only for high‐risk infants (such as congenital heart disease and premature birth) to align with current practice in Hong Kong [32]. Model inputs for palivizumab were based on published literature [11, 33]. Scenario 2 examined an extended duration of protection for nirsevimab, assuming 180 days of efficacy based on recently published randomized controlled trial data [34]. Scenario 3 applied QALY loss per RSV episode derived from a recent quality‐of‐life study [35], instead of calculating QALY loss using disutility values and event durations as in the base‐case analysis. Scenario 4 adopted a healthcare provider's perspective by including only direct medical costs (and excluding productivity losses and non‐medical costs). Scenario 5 excluded lifetime productivity losses due to RSV‐related deaths to address methodological concerns related to the human capital approach and potential double counting.

## 3 Results

### 3.1 Model Validation

The occurrence of clinical events simulated by the present model was validated with the incidence of events reported by other studies. In the no intervention group, the expected RSV‐LRTI associated hospitalization rate over 1 year was 213 per 10,000 infants, similar to the annual hospitalization rates in infants (156–233 per 10,000) reported by a Hong Kong 3‐year virologic study on RSV disease burden [36].

### 3.2 Base‐Case Analysis

In the base‐case analysis, all immunization strategies reduced the incidence of RSV‐LRTI infection, hospitalization, ICU admission, and death when compared to the no intervention group (Table 2). The nirsevimab catch‐up group achieved the lowest expected event rates among all strategies. The expected costs and QALY loss per 100,000 infants of the four strategies are presented in Table 3. The nirsevimab catch‐up group had the lowest QALY loss (38.82), followed by the nirsevimab year‐round group (45.71), the nirsevimab seasonal group (60.60), and the no intervention group (81.52).

**TABLE 2:** Expected clinical outcomes per 100,000 infants.

Strategy	RSV‐LRTI infection	Hospitalization	ICU admission	RSV‐related death
No intervention	3734	2132	51	0.90
Nirsevimab seasonal	2843	1491	34	0.63
Nirsevimab year‐round	2228	1025	22	0.43
Nirsevimab catch‐up	1944	838	17	0.35

Abbreviations: ICU: intensive care unit; LRTI, lower respiratory tract infections; RSV, respiratory syncytial virus.

**TABLE 3:** Base‐case analysis results on expected costs and QALY loss per 100,000 infants.

Strategy	Direct cost (USD)	Indirect cost^a^ (USD)	Total cost (USD)	QALY loss	ICER versus next less costly option	ICER versus no intervention
10% US cost (USD52)						
Nirsevimab catch‐up	8,384,346	2,420,382	10,804,729	38.82	—	**Dominant**
Nirsevimab year‐round	9,043,924	2,814,997	11,858,921	45.71	Dominated	**Dominant**
Nirsevimab seasonal	8,335,185	3,706,198	12,041,383	60.60	Dominated	**Dominant**
No intervention	7,562,816	4,976,027	12,538,843	81.52	Dominated	—
25% US cost (USD130)						
No intervention	7,562,816	4,976,027	12,538,843	81.52	—	—
Nirsevimab seasonal	12,724,473	3,706,198	16,430,671	60.60	Dominated	186,026
Nirsevimab catch‐up	16,179,140	2,420,382	18,599,522	38.82	**141,925**	**141,925**
Nirsevimab year‐round	16,840,174	2,814,997	19,655,171	45.71	Dominated	198,716
50% US cost (USD260)						
No intervention	7,562,816	4,976,027	12,538,843	81.52	—	—
Nirsevimab seasonal	20,039,955	3,706,198	23,746,153	60.60	Dominated	535,700
Nirsevimab catch‐up	29,170,462	2,420,382	31,590,844	38.82	446,148	446,148
Nirsevimab year‐round	29,833,924	2,814,997	32,648,921	45.71	Dominated	561,552

*Note:* Bold ICER, a strategy is cost‐effective with ICER < willingness‐to‐pay threshold (162,401 USD/QALY). ICER versus no vaccination = (Total cost_strategy_ − Total cost_no intervention_)/(QALY loss_no intervention_ − QALY loss_strategy_); ICER vs. next less costly option = (Total cost_strategy_ − Total cost next less costly_strategy_)/(QALY loss next less costly_strategy_ − QALY loss_strategy_).

Abbreviations: ICER, incremental cost per QALY gained; LRTI, lower respiratory tract infections; QALY, quality‐adjust life year; RSV, respiratory syncytial virus.

^a^ Indirect costs included productivity loss of caregiver time and premature death due to RSV‐associated mortality. The productivity loss over age 18–65 years in premature death was estimated to be USD 400,538 (discounted to current year at annual rate of 3%).

Using the no intervention group as the common comparator, all three immunization strategies resulted in lower total costs with QALY gains, dominating the no intervention group at the 10% US cost level (USD52). At the 25% US cost level (USD130), the nirsevimab catch‐up group achieved an ICER (141,925 USD/QALY) lower than the WTP threshold (162,401 USD/QALY saved). The ICERs of nirsevimab year‐round (198,716 USD/QALY) and nirsevimab seasonal groups (186,026 USD/QALY) were above the WTP threshold. At the 50% US cost level (USD260), all three strategies had higher costs with ICERs exceeding the threshold.

In a cost‐effectiveness ranking (each strategy vs. the next less costly option), the nirsevimab catch‐up groups dominated all three groups with the lowest QALY loss and total cost at the 10% US cost level. At the 25% and 50% cost levels, the nirsevimab year‐round and nirsevimab seasonal groups were dominated and therefore eliminated from further cost‐effectiveness analysis. The ICERs for the nirsevimab catch‐up group (vs. no intervention) were 141,925 USD/QALY at the 25% cost level and 446,148 USD/QALY at the 50% cost level, respectively.

### 3.3 Sensitivity Analyses

**FIGURE 2:**
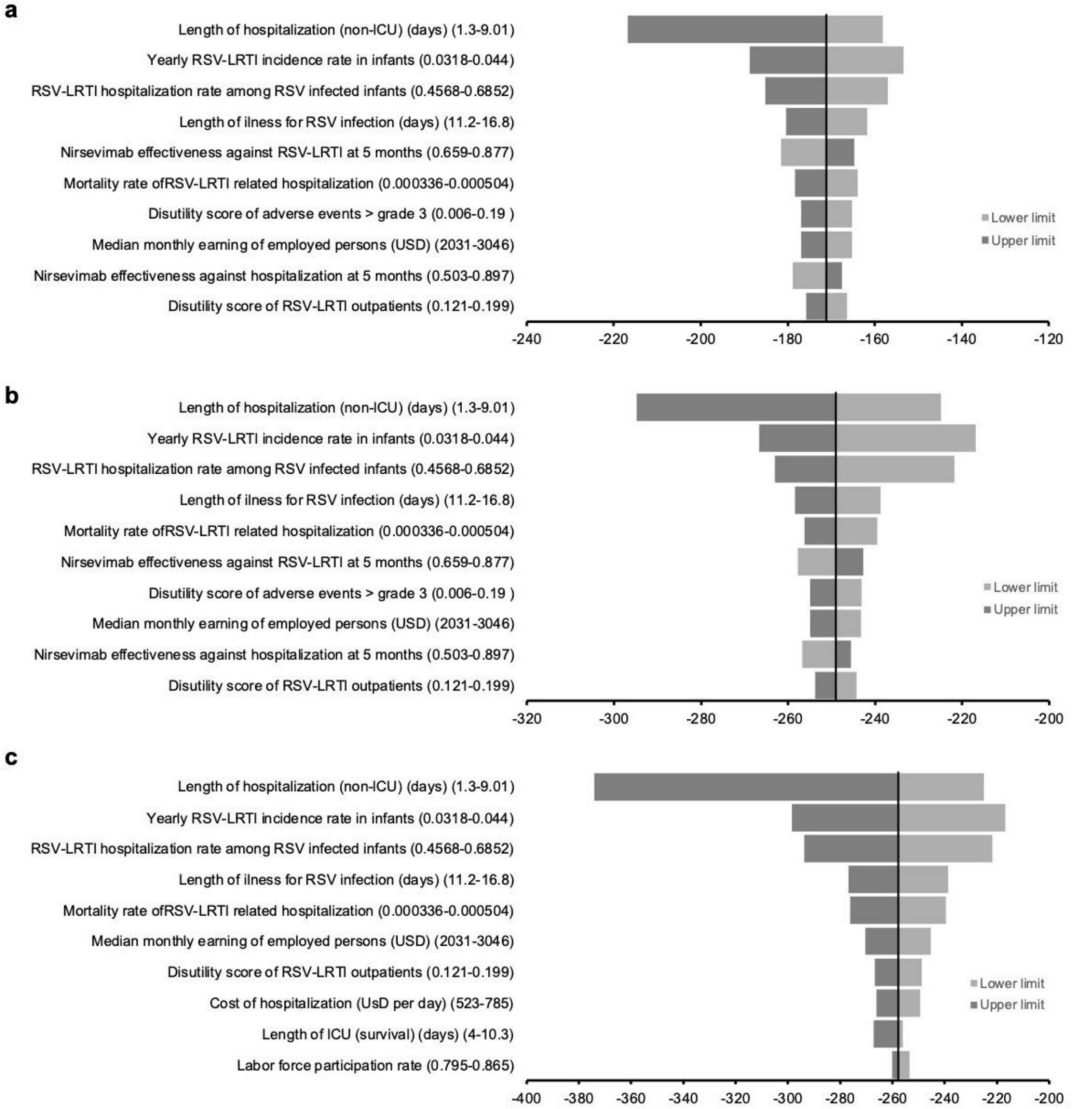
(a–c) Tornado diagrams of one‐way sensitivity analysis of top 10 influential parameters on net monetary benefit (NMB) for the preferred cost‐effective strategy at (a) 10% (USD52), (b) 25% (USD130), and (c) 50% (USD260) US cost levels. WTP, willingness‐to‐pay (162,401 USD/QALY) NMB = QALY loss*WTP threshold − Cost.

The top 10 influential factors on base‐case results found in the one‐way sensitivity analysis are illustrated in Figure 2a–c. At the 10% US cost level (USD52), the nirsevimab catch‐up group was the preferred cost‐effective strategy throughout the variation of all model inputs. At the 25% US cost level (USD130), threshold values were found for 6 influential factors: yearly RSV‐LRTI incidence rate in infants, RSV‐LRTI hospitalization rate among infected infants, length of hospitalization (survival), length of illness for RSV infection, monthly mortality rate of RSV‐infected infants during hospitalization, and nirsevimab effectiveness against RSV‐LRTI at 5 months. The preferred cost‐effective strategy changed from the nirsevimab catch‐up group to no intervention when the values of the 6 influential factors crossed the threshold values listed in Table 4. At the 50% cost level (USD260), the no intervention group was the preferred cost‐effective strategy across the variation of all model inputs. The drug cost threshold analysis showed that the nirsevimab catch‐up, year‐round, and seasonal strategies became cost‐effective (WTP threshold = 162,401 USD/QALY) (vs. no intervention) if the costs per dose of nirsevimab were less than USD134, USD113, and USD117, respectively (Figure S1).

**TABLE 4:** Threshold values of influential parameters in one‐way sensitivity analysis at 25% US cost level (USD130).

Parameters	Base‐case value	Threshold value^a^	Change of preferred cost‐effective strategy
Yearly RSV‐LRTI incidence rate in infants	0.0379	< 0.0356	No intervention
RSV‐LRTI hospitalization rate among infected infants	0.571	< 0.525	No intervention
Length of hospitalization (survival)	3	< 2.26	No intervention
Length of illness for RSV infection	14	< 11.47	No intervention
Monthly mortality rate of RSV‐infected infants during hospitalization	0.00042	< 0.00035	No intervention
Nirsevimab effectiveness against RSV‐LRTI at 5 months	0.795	< 0.681	No intervention

ARI, acute respiratory infection; LRTD, lower respiratory tract illness; RSV, respiratory syncytial virus.

^a^ The preferred cost‐effective strategy changed when the value of influential parameters crossed the threshold value.

**FIGURE 3:**
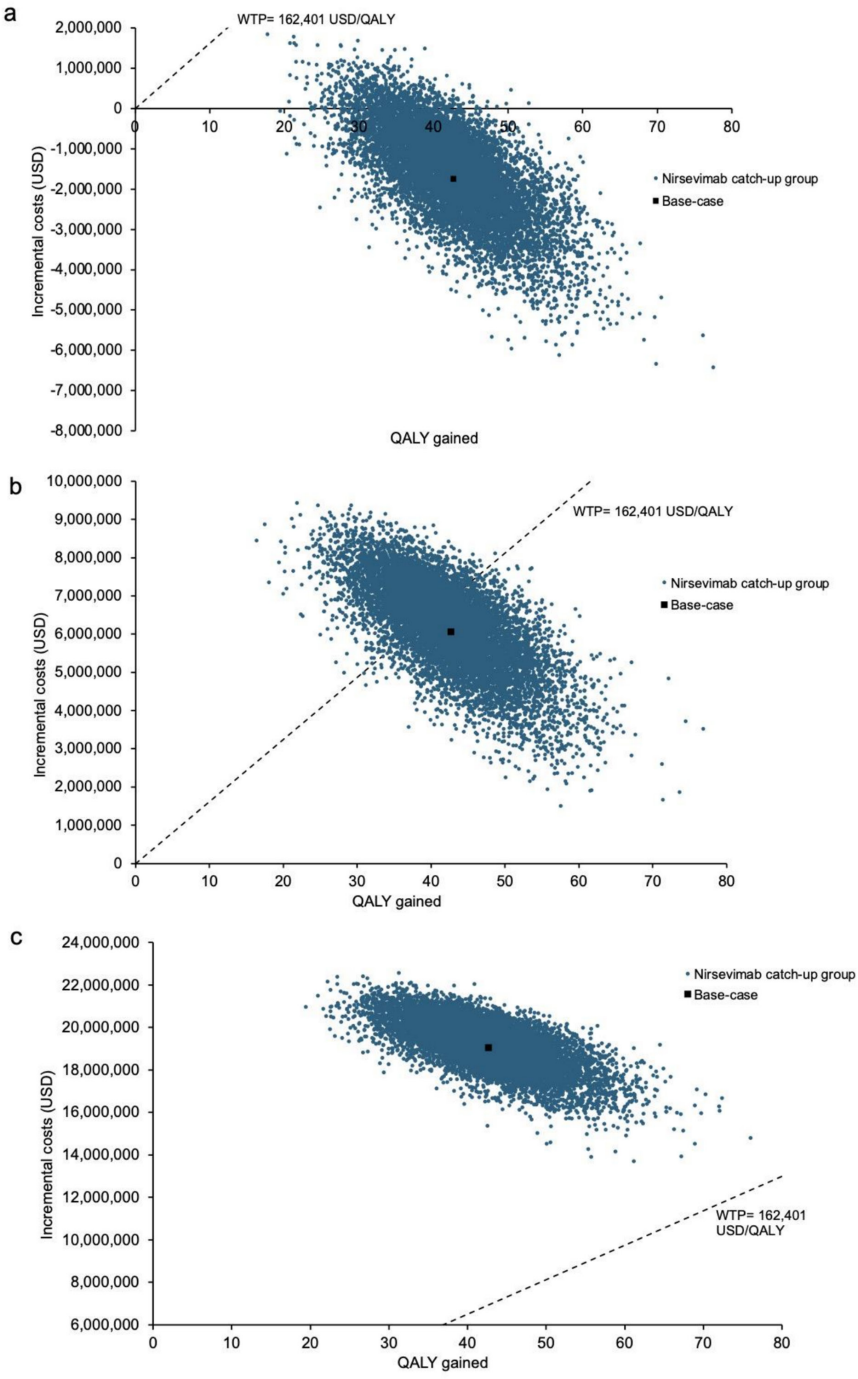
(a–c) Scatter plots of incremental costs and QALY gained by the nirsevimab catch‐up strategies versus no intervention in 10,000 Monte Carlo simulations at (a) 10% (USD52), (b) 25% (USD130), and (c) 50% (USD260) US cost levels. WTP, willingness‐to‐pay.

Scatter plots of the incremental total cost and QALYs gained by the nirsevimab catch‐up group versus no intervention at three cost levels are shown in Figure 3a–c. At the 10% cost level, 93.61% of the simulations are less costly and more effective, and 6.39% of the simulations are more effective at higher cost with ICER less than WTP thresholds. At the 25% and 50% cost levels, all simulations showed the nirsevimab catch‐up group gained QALYs at higher cost. The ICERs of the nirsevimab catch‐up group were below the WTP threshold in 60.59% and 0% of simulations at the 25% and 50% cost levels, respectively. The scatter plots of the nirsevimab year‐round and seasonal group compared with no intervention are presented in Figures S2 and S3. The mean (and 95% CI) incremental costs and QALYs gained by three immunization strategies versus no intervention in probabilistic sensitivity analysis are listed in Table S2.

**FIGURE 4:**
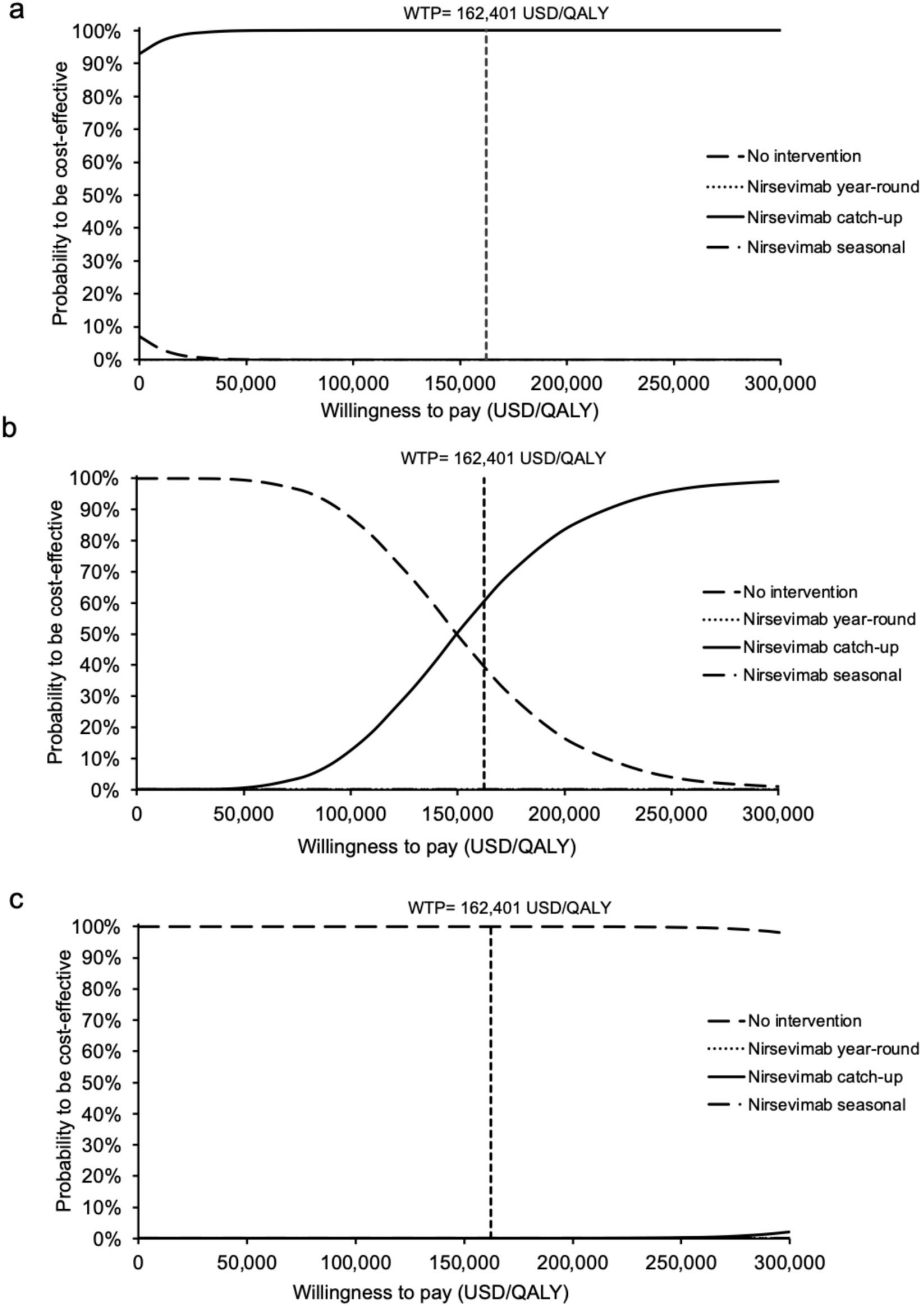
(a–c) Variation in the probability of all immunization strategies to be the preferred option against willingness‐to‐pay at (a) 10% (USD52), (b) 25% (USD130), and (c) 50% (USD260) US cost levels. WTP, willingness‐to‐pay.

The cost‐effectiveness acceptability curves of all strategies (vs. the next costly strategy) are shown in Figure 4a–c. The probability to be cost‐effective (at WTP = 162,401 USD/QALY) was 100% for the nirsevimab catch‐up group at the 10% US cost level. At 25% US cost level, the probability of being cost‐effective was 58.56%, 41.44%, 0%, and 0% (WTP = 162,401 USD/QALY) for the nirsevimab catch‐up, no intervention, nirsevimab year‐round, and nirsevimab seasonal group, respectively. The probability of the nirsevimab catch‐up group to be cost‐effective was lower than that of the no intervention group if the WTP threshold was < 49,545 USD/QALY. At 50% US cost level, the probability to be cost‐effective was 100% for the no intervention group.

**FIGURE 5:**
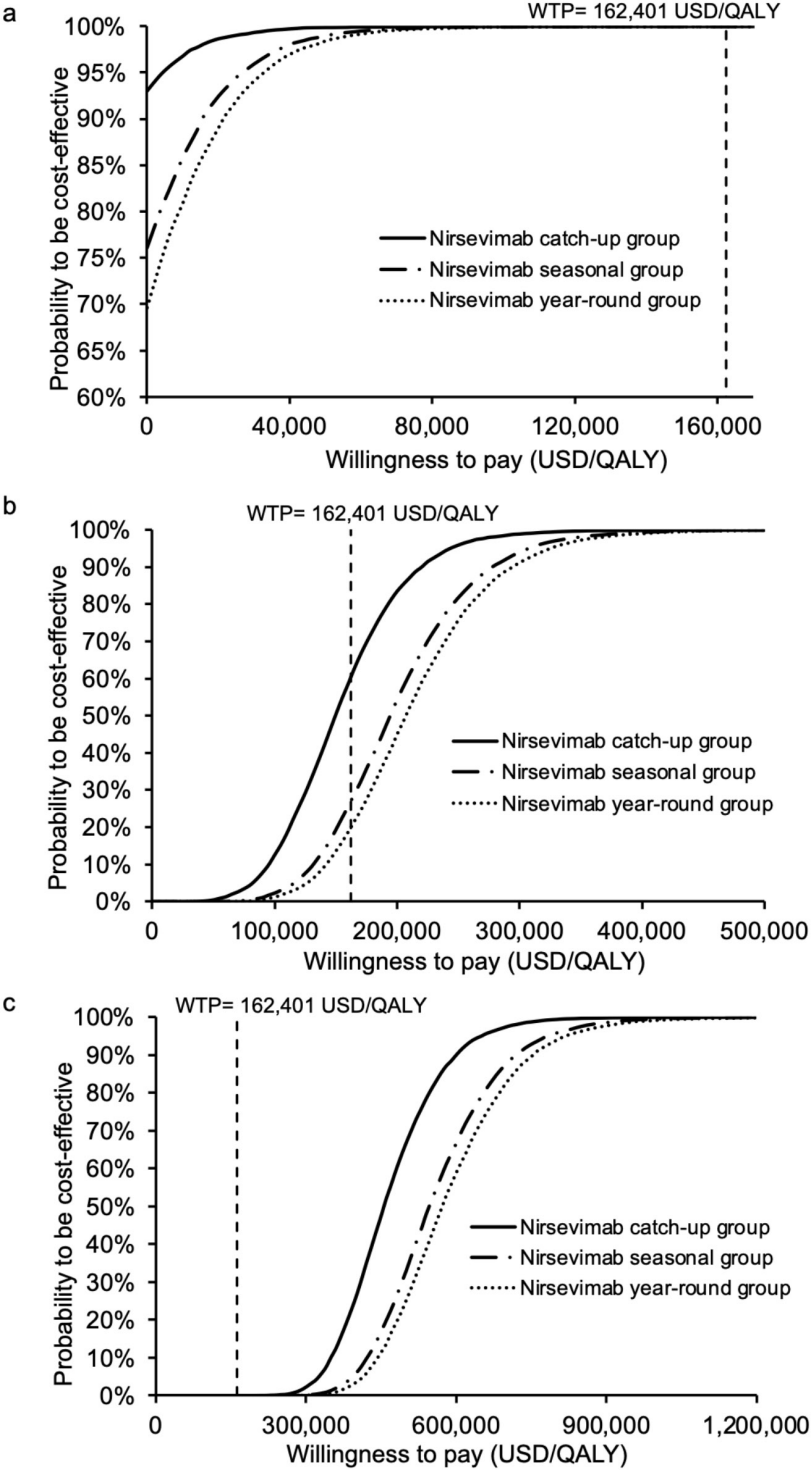
(a–c) Variation in probability of each nirsevmab immunization strategy versus no intervention to be cost‐effective against willingness‐to‐pay at (a) 10% (USD52), (b) 25% (USD130), and (c) 50% (USD260) US cost levels. WTP, willingness‐to‐pay.

The cost‐effectiveness acceptability curves of each nirsevimab strategy versus no intervention are shown in Figure 5a–c. At 10% US cost level, the probability to be cost‐effective was 100% for all three strategies compared with no intervention at the WTP = 162,401 USD/QALY. At 25% cost level, the probability to be cost‐effective was 60.43%, 26.94%, and 19.86% for the nirsevimab catch‐up, nirsevimab seasonal, and nirsevimab year‐round group, respectively. At 50% US cost level, the probability to be cost‐effective was 0% for all three strategies.

### 3.4 Scenario Analyses

In Scenario 1, palivizumab resulted in higher QALY loss than the nirsevimab strategies. It was dominated when compared to the next costly option and was not accepted as cost‐effective (with ICER > WTP) when compared with no intervention (Table S3). In Scenario 2, extending nirsevimab protection to 180 days improved the cost‐effectiveness (lowering the total costs and QALY loss) of all nirsevimab strategies (Table S4). An alternative QALY loss was applied in Scenario 3 and did not alter the overall results (Table S5). The perspective of the healthcare provider was adopted in Scenario 4 and resulted in increased ICERs across all strategies (Table S6). Scenario 5 (excluding productivity loss due to death) showed slightly varied ICERs but did not change the overall results (Table S7).

## 4 Discussion

This study is the first to assess the cost‐effectiveness of different immunization strategies of nirsevimab, a long‐acting monoclonal antibody, for preventing RSV infection in infants in Hong Kong. Three strategies (nirsevimab catch‐up, nirsevimab year‐round, and nirsevimab seasonal) were assessed at various US cost levels to provide detailed economic evidence for decision making, with potential relevance for other regions experiencing similar RSV patterns. The base‐case analysis indicated that all three immunization strategies reduced RSV–LRTI‐related event rates and increased QALYs, with the nirsevimab catch‐up strategy ranking first, followed by the nirsevimab year‐round, nirsevimab seasonal, and no immunization. The cost‐effectiveness of nirsevimab strategies was highly sensitive to cost. At 10% of the US cost, all three immunization strategies became cost‐saving compared to no intervention. At 50% of the US cost, none of the nirsevimab strategies were cost‐effective. The findings of probabilistic sensitivity analysis and scenario analysis supported the robustness of base‐case results at 10% and 50% cost levels.

At 25% of the US cost, the nirsevimab catch‐up strategy was cost‐effective (vs. no intervention or vs. the next less costly option), with an ICER of 141,925 USD/QALY. Model inputs of RSV‐LRTI incidence, RSV‐LRTI related clinical consequences, and the effectiveness of nirsevimab were identified as influential factors with thresholds in the one‐way sensitivity analysis. At a lower RSV‐LRTI incidence rate, the number of infections and RSV‐LRTI related hospitalizations prevented by the nirsevimab catch‐up strategy (vs. no intervention) were reduced, narrowing the QALY saved by nirsevimab. The cost‐saving generated by the marginal reduction of infected cases and hospitalization was therefore narrowed, leading to increased incremental cost. As a result, the ICER increased at a lower RSV‐LRTI incidence rate, and the nirsevimab catch‐up strategy became not cost‐effective when the RSV‐LRTI incidence rate dropped below 0.0356. Variation of RSV‐LRTI related consequences (hospitalization rate, mortality, length of hospitalization, and duration of illness) was shown to influence the cost‐effectiveness outcomes. At lower hospitalization or mortality, QALY saved by the nirsevimab catch‐up strategy was narrowed. Shorter duration of hospitalization or illness narrowed the cost‐saving generated by the nirsevimab catch‐up strategy. Similar to the effect of a lower incidence rate, the ICERs of the nirsevimab catch‐up strategy were increased. Declining nirsevimab effectiveness led to fewer cases and hospitalizations prevented, thus lowering both QALY saved and cost‐saving. The ICERs of the nirsevimab catch‐up strategy increased as nirsevimab effectiveness declined. At the 10% and 50% US cost levels, the key influential factors shown by one‐way sensitivity analysis were consistently related to RSV‐LRTI incidence rate, RSV infection‐related consequences, and nirsevimab effectiveness, despite no threshold value being identified. The sensitivity analysis findings illustrated the combined impact of disease burden and immunization effectiveness on the cost‐effectiveness of nirsevimab. In the present model, the high drug cost of nirsevimab emerged as a significant factor in the cost‐effectiveness acceptance of nirsevimab. Our drug cost threshold analysis indicated that threshold nirsevimab costs for nirsevimab catch‐up, year‐round, and seasonal strategies to become cost‐effective.

One prior cost‐effectiveness study compared year‐round, seasonal, and catch‐up programs of maternal vaccination and infant monoclonal antibody [37]. The expected reduction in RSV‐LRTI associated hospitalization rate was up to 72.2% in the monoclonal antibody catch‐up strategy for infants. In our model, the expected hospitalization rate of newborns was reduced by 60.7% for the nirsevimab catch‐up group and was similar to the previous findings [37]. A recent cost‐effectiveness analysis evaluated the monoclonal antibody against RSV versus no intervention in infants across six European countries [38]. The reported findings suggested that the cost‐effectiveness findings were sensitive to the country‐specific WTP threshold, monoclonal antibody cost, and age‐specific RSV‐related hospitalizations. In the present study, our results also consistently showed that drug cost and RSV‐related hospitalization rate were influential factors in the cost‐effectiveness results. In addition, our study assessed the cost‐effectiveness of nirsevimab across three price scenarios (10%, 25%, and 50% of the US price), offering evidence to support pricing and reimbursement decisions. Together with the one‐way sensitivity analysis findings, key parameters and corresponding thresholds were provided for policymakers to adjust pricing, define target populations, and prioritize resources based on local epidemiology and budget.

The use of 3 × GDP per capita as the WTP threshold is subject to debate [39] and some alternative approaches have been proposed in the literature [40]. In the absence of an accepted WTP threshold in Hong Kong, the present study applied 3 × GDP per capita as the WTP threshold to allow comparison with published cost‐effectiveness studies [21, 41, 42]. The selection of a WTP threshold for interpretation of cost‐effectiveness should be context‐specific and consider the type of intervention, target population, and local decision‐making priorities.

Although nirsevimab has been registered in Hong Kong, it is classified as a self‐financed item outside the government drug formulary, potentially limiting access and reducing uptake. Our findings suggest that nirsevimab could be cost‐effective in Hong Kong at a low‐price level. The uptake of nirsevimab is expected to be low when public funding or subsidy is not available, and population‐level effectiveness is likely to be lower than the model outcomes (assuming 100% coverage). To support implementation, policy actions such as price negotiation, targeted subsidy for high‐risk groups, and integration into existing maternal and child health services should be considered. In addition to monoclonal antibodies, maternal immunization with RSV vaccine is another preventive approach. The present study evaluated, at the time when the infant is born to a woman who did not receive maternal RSV vaccination, the cost‐effectiveness of offering nirsevimab at different timings (all year around, during RSV season, or during RSV season (with catch up)). The present analysis therefore did not include maternal RSV vaccination as a comparator. Both monoclonal antibodies and maternal immunization strategies offer passive protection during early infancy but differ in administration timing. Maternal vaccination may be easier to integrate into antenatal care but could provide less protection for preterm infants or when uptake is suboptimal. Nirsevimab offers direct protection regardless of maternal status, and could be administered to those who did not receive maternal RSV vaccination [43]. The choice between these strategies should consider clinical recommendations, individual clinical characteristics, and accessibility.

There were some limitations in the study associated with uncertainties of model inputs. Infants with heart disease or lung disease, preterm infants, and infants under 6 months are at higher risk of hospitalization following RSV infection. However, due to the absence of local data stratified by risk group, the model applied an average RSV‐LRTI hospitalization rate across the infant population and did not distinguish between high‐risk and low‐risk subgroups. The differential cost‐effectiveness of nirsevimab immunization by risk status is therefore not evaluated. As high‐risk infants experience a greater disease burden, the cost‐effectiveness of nirsevimab in this subgroup may be more favorable than the estimates in the general population. When stratified and representative local data become available, subgroup analyses are warranted to provide more targeted insights for policy decisions. The local data of RSV‐LRTD hospitalization rate among infected cases was not available, and we therefore adopted the estimates from other high‐income countries and distributed them monthly using Hong Kong‐specific surveillance data. While this approximation captured local seasonality, it may not fully reflect the local RSV hospitalizations and introduced uncertainty into the model estimates. The nirsevimab effectiveness was retrieved from overseas studies and might affect the generalizability to local patients. Rigorous sensitivity analyses were performed to evaluate the influence of model inputs on result robustness. Lastly, the model assumed 100% coverage of nirsevimab among eligible newborns, and the impact of varying coverage rates on cost‐effectiveness was not assessed.

## 5 Conclusions

All nirsevimab strategies reduced RSV‐LRTI associated event rates and improved QALYs of infants. At 10% US drug cost level (USD52), the nirsevimab catch‐up strategy was cost‐saving. At 25% US drug cost level (USD130), the nirsevimab catch‐up strategy was the preferred cost‐effective option, subject to RSV annual incidence rate, RSV‐LRTI related consequences, and effectiveness of nirsevimab against RSV‐LRTI. At 50% US drug cost level (USD260), all nirsevimab strategies were not accepted as cost‐effective.

## Author Contributions

**Yingcheng Wang:** conceptualization, methodology, validation, formal analysis, investigation, data curation, writing – original draft, software. **Mingjun Rui:** methodology, software, validation, formal analysis, data curation, investigation. **Qiran Wei:** validation, formal analysis, investigation, data curation. **Ting Fan Leung:** conceptualization, writing – review and editing. **Joyce H. S. You:** conceptualization, supervision, validation, writing – review and editing.

## Conflicts of Interest

The authors declare no conflicts of interest.

## Supporting information


**Data S1:** Supplementary Information.

**Figure S1:** Nirsevimab price (per dose) thresholds against willingness‐to‐pay for different immunization strategies (vs. no intervention).

**Figure S2:** (a–c) Scatter plots of incremental costs and QALY gained by the nirsevimab year‐round strategies versus no intervention in 10,000 Monte Carlo simulations at (a) 10% (USD52), (b) 25% (USD130), and (c) 50% (USD260) US price levels; WTP: Willingness‐to‐pay.

**Figure S3:** (a–c) Scatter plots of incremental costs and QALY gained by the nirsevimab seasonal strategies versus no intervention in 10,000 Monte Carlo simulations at (a) 10% (USD52), (b) 25% (USD130), and (c) 50% (USD260) US price levels; WTP: Willingness‐to‐pay.

**Table S1:** RSV‐LRTI related event incidence rate in 12 calendar months.

**Table S2:** Incremental costs and QALY gained by three immunization strategies versus no intervention in probabilistic sensitivity analysis.

**Table S3:** Scenario 1 (with palivizumab comparator) results on expected costs and QALY loss per 100,000 infants.

**Table S4:** Scenario 2 (extended 180‐day nirsevimab efficacy) results on expected costs and QALY loss per 100,000 infants.

**Table S5:** Scenario 3 (alternative QALY loss source) results on expected costs and QALY loss per 100,000 infants.

**Table S6:** Scenario 4 (healthcare provider's perspective) results on expected costs and QALY loss per 100,000 infants.

**Table S7:** Scenario 5 (excluded lifetime productivity losses due to death) results on expected costs and QALY loss per 100,000 infants.

## Data Availability

All the data related to this study are available in the manuscript.
