# Supplementary material for: Cost‐Effectiveness Analysis of Nirsevimab for Respiratory Syncytial Virus Disease Prevention in Newborns of Hong Kong
Source: Influenza Other Respir Viruses. 2025 Oct 1;19(10):e70153. doi: 10.1111/irv.70153 (PMC12485666; doi:10.1111/irv.70153)
Supplement: Supplementary file 2 — Figure S1: Nirsevimab price (per dose) thresholds against willingness‐to‐pay for different immunization strategies (vs. no intervention). [file IRV-19-e70153-s010.docx]

**Supplementary Materials**

**
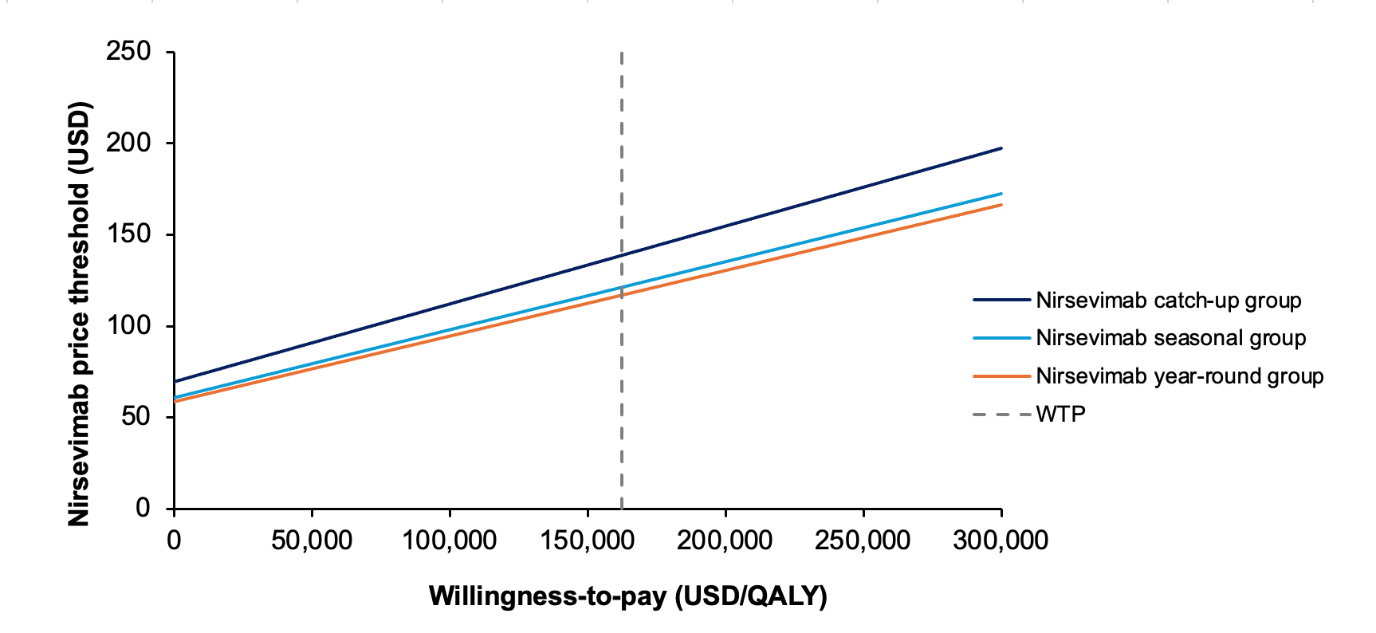
**

**Figure S1** Nirsevimab price (per dose) thresholds against willingness-to-pay for different immunization strategies (versus no intervention)
