# Supplementary material for: Cost‐Effectiveness Analysis of Nirsevimab for Respiratory Syncytial Virus Disease Prevention in Newborns of Hong Kong
Source: Influenza Other Respir Viruses. 2025 Oct 1;19(10):e70153. doi: 10.1111/irv.70153 (PMC12485666; doi:10.1111/irv.70153)
Supplement: Supplementary file 3 — Figure S2: (a–c) Scatter plots of incremental costs and QALY gained by the nirsevimab year‐round strategies versus no intervention in 10,000 Monte Carlo simulations at (a) 10% (USD52), (b) 25% (USD130), and (c) 50% (USD260) US price levels; WTP: Willingness‐to‐pay. [file IRV-19-e70153-s009.docx]

**Supplementary Materials**

**
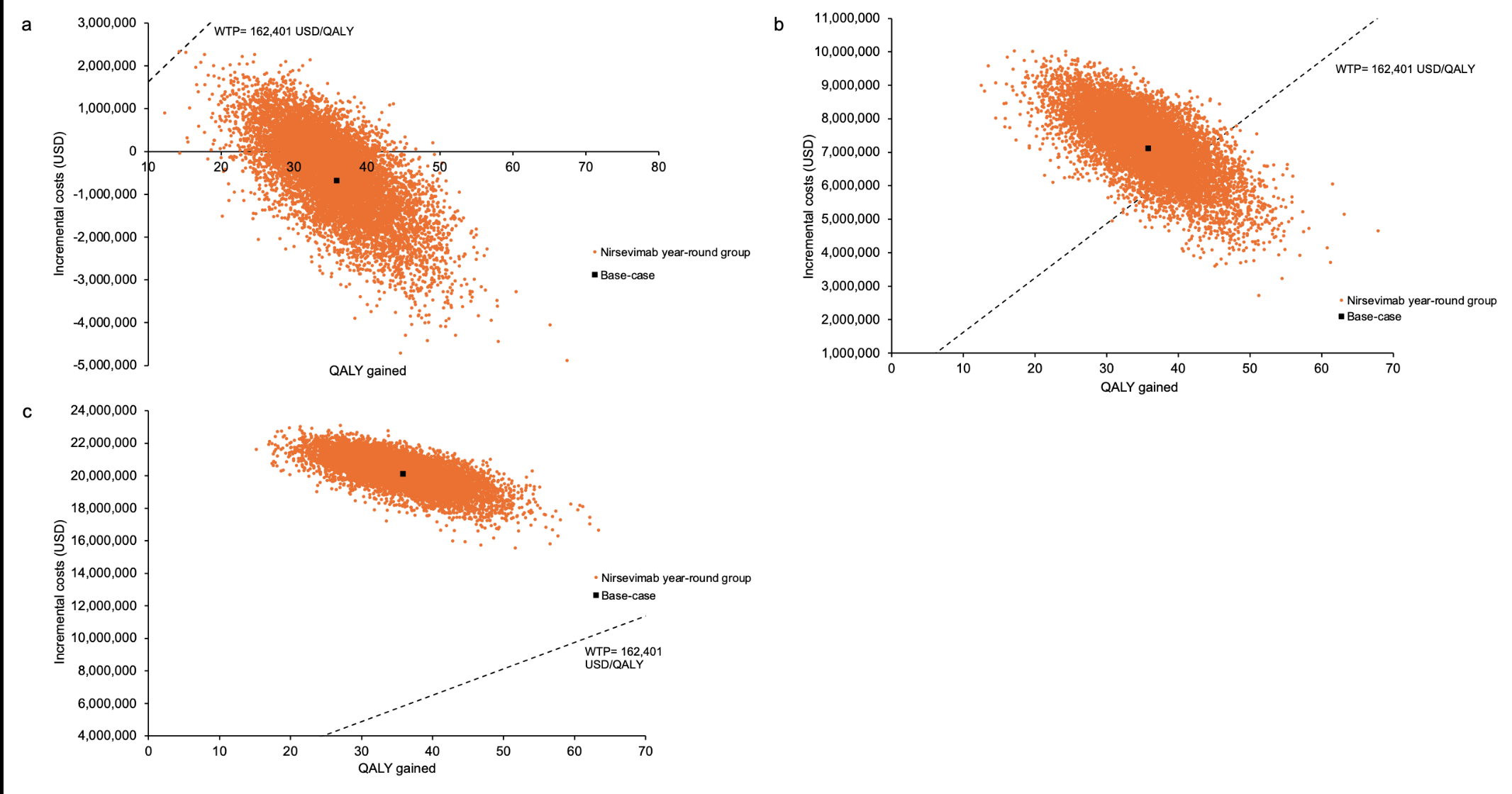
**

**Figure S2a-c.** Scatter plots of incremental costs and QALY gained by the nirsevimab year-round strategies versus no intervention in 10,000 Monte Carlo simulations at (a) 10% (USD52), (b) 25% (USD130), and (c) 50% (USD260) US price levels; WTP: Willingness-to-pay
