# Supplementary material for: Cost‐Effectiveness Analysis of Nirsevimab for Respiratory Syncytial Virus Disease Prevention in Newborns of Hong Kong
Source: Influenza Other Respir Viruses. 2025 Oct 1;19(10):e70153. doi: 10.1111/irv.70153 (PMC12485666; doi:10.1111/irv.70153)
Supplement: Supplementary file 5 — Table S1: RSV‐LRTI related event incidence rate in 12 calendar months. [file IRV-19-e70153-s007.docx]

**Supplementary Materials**

**Yearly RSV-LRTI incidence and relative RSV-LRTI incidence ratio of in-season versus off-season**

The annual number (963) of RSV-related hospitalization in infants and the monthly proportion of hospitalized cases in 12 calendar months were reported by a cost-effectiveness analysis on of monoclonal antibody (which used the Hong Kong RSV sentinel surveillance data in 1998-2015) [1]. The RSV-related hospitalization number in each calendar month was calculated using the annul number of hospitalization (963) and the monthly proportion of hospitalization [1]. RSV-related hospitalization rate in infants (monthly per population) was estimated by the RSV-related hospitalization number in each calendar month and the corresponding infant population [2]. RSV-LRTI incidence rate in infants (monthly per population) was estimated using the RSV-related hospitalization rate in infants (monthly per population) and hospitalization rate among infected infants (57.1%) [3]. The yearly RSV-LRTI incidence rate in infants per population (3.79%) was the summation of the RSV-LRTI incidence rate in infants (monthly per population) of 12 calendar months. The average RSV-LRTI incidence rates in infants (monthly per population) were 0.4373% in peak season (March to September) and 0.1456% in off-season (October to February). The relative RSV-LRTI incidence ratio of in-season versus off-season was (0.4373%/0.1456%) 3.01. The estimations are shown in **Table S1**.

**Table S1 RSV-LRTI related event incidence rate in 12 calendar months**

| **Calendar month** | **Monthly proportion of hospitalization [1]** | **RSV-related hospitalization number in each calendar month^†^** | **Infant population [2]** | **RSV-related hospitalization rate in infants (monthly per population)^‡^** | **RSV-LRTI incidence rate in infants (monthly per population)^§^** |
| --- | --- | --- | --- | --- | --- |
| January | 0.038 | 37 | 47600 | 0.00077 | 0.00135 |
| February | 0.061 | 59 | 42198 | 0.00140 | 0.00244 |
| March | 0.132 | 127 | 45449 | 0.00280 | 0.00490 |
| April | 0.151 | 146 | 40484 | 0.00360 | 0.00629 |
| May | 0.106 | 102 | 43831 | 0.00233 | 0.00408 |
| June | 0.089 | 86 | 43082 | 0.00199 | 0.00349 |
| July | 0.113 | 109 | 44321 | 0.00246 | 0.00430 |
| August | 0.108 | 104 | 46062 | 0.00226 | 0.00396 |
| September | 0.097 | 94 | 45589 | 0.00205 | 0.00359 |
| October | 0.047 | 45 | 49763 | 0.00091 | 0.00160 |
| November | 0.023 | 22 | 51291 | 0.00043 | 0.00076 |
| December | 0.033 | 32 | 49346 | 0.00065 | 0.00113 |

RSV: Respiratory syncytial virus; LRTI: lower respiratory tract infections

†: RSV-related hospitalization number in each calendar month = 963 * Monthly proportion of hospitalization

‡: RSV-related hospitalization rate in infants (monthly per population) = RSV-related hospitalization number in each calendar month / month-specific infant population

§: RSV-LRTI incidence rate in infants (monthly per population) = RSV-related hospitalization rate in infants (monthly per population) / hospitalization rate among infected infants (0.571)

**Reference**

[1] WU Peng CBJ, CHIU Shiu-seng Susan, WONG Oi Ling Irene, YEUNG Kwan Yee Wilson. Cost-effectiveness of prophylaxis with palivizumab in high-risk children in Hong Kong 2017 [cited 2024 Nov 11]. Available from: <https://rfs2.healthbureau.gov.hk/search/#/fundedsearch/projectdetail?id=2520&lang=en>

[2] Census and Statistics Department. Table 110-01002 : Population by Sex and Age [cited 2025 Feb 9]. Available from: <https://www.censtatd.gov.hk/en/web_table.html?id=110-01002>

[3] Li Y, Wang X, Blau DM, et al. Global, regional, and national disease burden estimates of acute lower respiratory infections due to respiratory syncytial virus in children younger than 5 years in 2019: a systematic analysis. The Lancet. 2022;399(10340):2047-2064. doi:10.1016/S0140-6736(22)00478-0
