# Supplementary material for: Cost‐Effectiveness Analysis of Nirsevimab for Respiratory Syncytial Virus Disease Prevention in Newborns of Hong Kong
Source: Influenza Other Respir Viruses. 2025 Oct 1;19(10):e70153. doi: 10.1111/irv.70153 (PMC12485666; doi:10.1111/irv.70153)
Supplement: Supplementary file 6 — Table S2: Incremental costs and QALY gained by three immunization strategies versus no intervention in probabilistic sensitivity analysis. [file IRV-19-e70153-s001.docx]

**Supplementary Materials**

**Table S2.** Incremental costs and QALY gained by three immunization strategies versus no intervention in probabilistic sensitivity analysis

| **Strategy** | **QALY gained, mean (95% CI)** | **Incremental costs (USD), mean (95% CI)** |
| --- | --- | --- |
| 10% US price (USD52) |  |  |
| Nirsevimab year-round | 35.57 (35.45-35.69) | -536,553 (-555,538- -517,569) |
| Nirsevimab catch-up | 42.39 (42.26-42.53) | -1,571,135 (-1,593,324- -1,548,945) |
| Nirsevimab seasonal | 20.75 (20.67-20.83) | -411,167 (-422,302- -400,031) |
| 25% US price (US130) |  |  |
| Nirsevimab year-round | 35.52 (35.40-35.64) | 7,255,980 (7,236,895-7,275,066) |
| Nirsevimab catch-up | 42.34 (42.40-42.48) | 6,218,201 (6,195,874-6,240,528) |
| Nirsevimab seasonal | 20.74 (20.66-20.82) | 3,972,359 (3,959,255-3,985,463) |
| 50% US price (USD260) |  |  |
| Nirsevimab year-round | 35.60 (35.48-35.72) | 20,237,041 (20,218,058-20,256,024) |
| Nirsevimab catch-up | 42.44 (42.31-42.58) | 19,193,689 (19,171,523-19,215,855) |
| Nirsevimab seasonal | 20.77 (20.69-20.85) | 11,270,071 (11,247,675-11,292,467) |
