# Supplementary material for: Cost‐Effectiveness Analysis of Nirsevimab for Respiratory Syncytial Virus Disease Prevention in Newborns of Hong Kong
Source: Influenza Other Respir Viruses. 2025 Oct 1;19(10):e70153. doi: 10.1111/irv.70153 (PMC12485666; doi:10.1111/irv.70153)
Supplement: Supplementary file 7 — Table S3: Scenario 1 (with palivizumab comparator) results on expected costs and QALY loss per 100,000 infants. [file IRV-19-e70153-s006.docx]

**Supplementary Materials**

**Table S3 Scenario 1 (with palivizumab comparator) results on expected costs and QALY loss per 100,000 infants**

| Strategy | Direct cost (USD) | Indirect cost (USD) | Total cost (USD) | QALY loss | ICER vs. next less costly option | ICER vs. no Intervention |
| --- | --- | --- | --- | --- | --- | --- |
| 10% US cost (USD52) |  |  |  |  |  |  |
| Nirsevimab catch-up | 8,384,346 | 2,420,382 | 10,804,729 | 38.82 | - | **dominant** |
| Nirsevimab year-round | 9,043,924 | 2,814,997 | 11,858,921 | 45.71 | dominated | **dominant** |
| Nirsevimab seasonal | 8,335,185 | 3,706,198 | 12,041,383 | 60.60 | dominated | **dominant** |
| No intervention | 7,562,816 | 4,976,027 | 12,538,843 | 81.52 | dominated | - |
| Palivizumab | 16,487,189 | 4,739,412 | 21,226,601 | 71.14 | dominated | 836,895 |
| 25% US cost (USD130) |  |  |  |  |  |  |
| No intervention | 7,562,816 | 4,976,027 | 12,538,843 | 81.52 | - | - |
| Nirsevimab seasonal | 12,724,473 | 3,706,198 | 16,430,671 | 60.60 | dominated | 186,026 |
| Nirsevimab catch-up | 16,179,140 | 2,420,382 | 18,599,522 | 38.82 | **141,925** | **141,925** |
| Nirsevimab year-round | 16,840,174 | 2,814,997 | 19,655,171 | 45.71 | dominated | 198,716 |
| Palivizumab | 16,487,189 | 4,739,412 | 21,226,601 | 71.14 | dominated | 836,895 |
| 50% US cost (USD260) |  |  |  |  |  |  |
| No intervention | 7,562,816 | 4,976,027 | 12,538,843 | 81.52 | - | - |
| Palivizumab | 16,487,189 | 4,739,412 | 21,226,601 | 71.14 | dominated | 836,895 |
| Nirsevimab seasonal | 20,039,955 | 3,706,198 | 23,746,153 | 60.60 | dominated | 535,700 |
| Nirsevimab catch-up | 29,170,462 | 2,420,382 | 31,590,844 | 38.82 | 446,148 | 446,148 |
| Nirsevimab year-round | 29,833,924 | 2,814,997 | 32,648,921 | 45.71 | dominated | 561,552 |

RSV: Respiratory Syncytial Virus; LRTI: lower respiratory tract infections; QALY; quality-adjust life year. ICER: incremental cost per QALY gained; ICER vs. next less costly option= (Total cost _strategy_- Total cost next less costly _strategy_)/ (QALY loss next less costly _strategy_- QALY loss _strategy_); ICER vs. no vaccination = (Total cost _strategy_- Total cost _no intervention_)/(QALY loss _no intervention_- QALY loss _strategy_). Bold ICER: A strategy is cost-effective with ICER < willingness-to-pay threshold (162,401 USD/QALY).
