# Supplementary material for: Cost‐Effectiveness Analysis of Nirsevimab for Respiratory Syncytial Virus Disease Prevention in Newborns of Hong Kong
Source: Influenza Other Respir Viruses. 2025 Oct 1;19(10):e70153. doi: 10.1111/irv.70153 (PMC12485666; doi:10.1111/irv.70153)
Supplement: Supplementary file 8 — Table S4: Scenario 2 (extended 180‐day nirsevimab efficacy) results on expected costs and QALY loss per 100,000 infants. [file IRV-19-e70153-s005.docx]

**Supplementary Materials**

**Table S4 Scenario 2 (extended 180-day nirsevimab efficacy) results on expected costs and QALY loss per 100,000 infants**

| Strategy | Direct cost (USD) | Indirect cost (USD) | Total cost (USD) | QALY loss | ICER vs. next less costly option | ICER vs. no Intervention |
| --- | --- | --- | --- | --- | --- | --- |
| 10% US cost (USD52) |  |  |  |  |  |  |
| Nirsevimab catch-up | 8,255,663 | 2,392,837 | 10,648,500 | 37.81 | - | **dominant** |
| Nirsevimab year-round | 8,910,177 | 2,786,724 | 11,696,901 | 44.67 | dominated | **dominant** |
| Nirsevimab seasonal | 8,276,437 | 3,693,378 | 11,969,815 | 60.15 | dominated | **dominant** |
| No intervention | 7,562,816 | 4,976,027 | 12,538,843 | 81.52 | dominated | - |
| 25% US cost (USD130) |  |  |  |  |  |  |
| No intervention | 7,562,816 | 4,976,027 | 12,538,843 | 81.52 | - | - |
| Nirsevimab seasonal | 12,665,726 | 3,693,378 | 16,359,104 | 60.15 | dominated | 178,785 |
| Nirsevimab catch-up | 16,050,456 | 2,392,837 | 18,443,293 | 37.81 | **135,086** | **135,086** |
| Nirsevimab year-round | 16,706,427 | 2,786,724 | 19,493,151 | 44.67 | dominated | 188,726 |
| 50% US cost (USD260) |  |  |  |  |  |  |
| No intervention | 7,562,816 | 4,976,027 | 12,538,843 | 81.52 | - | - |
| Nirsevimab seasonal | 19,981,207 | 3,693,378 | 23,674,585 | 60.15 | dominated | 521,144 |
| Nirsevimab catch-up | 29,041,779 | 2,392,837 | 31,434,616 | 37.81 | 432,309 | 432,309 |
| Nirsevimab year-round | 29,700,177 | 2,786,724 | 32,486,901 | 44.67 | dominated | 541,350 |

RSV: Respiratory Syncytial Virus; LRTI: lower respiratory tract infections; QALY; quality-adjust life year. ICER: incremental cost per QALY gained; ICER vs. next less costly option= (Total cost _strategy_- Total cost next less costly _strategy_)/ (QALY loss next less costly _strategy_- QALY loss _strategy_); ICER vs. no vaccination = (Total cost _strategy_- Total cost _no intervention_)/(QALY loss _no intervention_- QALY loss _strategy_). Bold ICER: A strategy is cost-effective with ICER < willingness-to-pay threshold (162,401 USD/QALY).
