# Supplementary material for: Cost‐Effectiveness Analysis of Nirsevimab for Respiratory Syncytial Virus Disease Prevention in Newborns of Hong Kong
Source: Influenza Other Respir Viruses. 2025 Oct 1;19(10):e70153. doi: 10.1111/irv.70153 (PMC12485666; doi:10.1111/irv.70153)
Supplement: Supplementary file 9 — Table S5: Scenario 3 (alternative QALY loss source) results on expected costs and QALY loss per 100,000 infants. [file IRV-19-e70153-s002.docx]

**Supplementary Materials**

**Table S5 Scenario 3 (alternative QALY loss source) results on expected costs and QALY loss per 100,000 infants**

| Strategy | Direct cost (USD) | Indirect cost (USD) | Total cost (USD) | QALY loss | ICER vs. next less costly option | ICER vs. no Intervention |
| --- | --- | --- | --- | --- | --- | --- |
| 10% US cost (USD52) |  |  |  |  |  |  |
| Nirsevimab catch-up | 8,384,346 | 2,420,382 | 10,804,729 | 38.52 | - | **dominant** |
| Nirsevimab year-round | 9,043,924 | 2,814,997 | 11,858,921 | 45.69 | dominated | **dominant** |
| Nirsevimab seasonal | 8,335,185 | 3,706,198 | 12,041,383 | 61.51 | dominated | **dominant** |
| No intervention | 7,562,816 | 4,976,027 | 12,538,843 | 83.59 | dominated | - |
| 25% US cost (USD130) |  |  |  |  |  |  |
| No intervention | 7,562,816 | 4,976,027 | 12,538,843 | 83.59 | - | - |
| Nirsevimab seasonal | 12,724,473 | 3,706,198 | 16,430,671 | 61.51 | dominated | 176,195 |
| Nirsevimab catch-up | 16,179,140 | 2,420,382 | 18,599,522 | 38.52 | **134,456** | **134,456** |
| Nirsevimab year-round | 16,840,174 | 2,814,997 | 19,655,171 | 45.69 | dominated | 187,756 |
| 50% US cost (USD260) |  |  |  |  |  |  |
| No intervention | 7,562,816 | 4,976,027 | 12,538,843 | 83.59 | - | - |
| Nirsevimab seasonal | 20,039,955 | 3,706,198 | 23,746,153 | 61.51 | dominated | 507,391 |
| Nirsevimab catch-up | 29,170,462 | 2,420,382 | 31,590,844 | 38.52 | 422,667 | 422,667 |
| Nirsevimab year-round | 29,833,924 | 2,814,997 | 32,648,921 | 45.69 | dominated | 530,582 |

RSV: Respiratory Syncytial Virus; LRTI: lower respiratory tract infections; QALY; quality-adjust life year. ICER: incremental cost per QALY gained; ICER vs. next less costly option= (Total cost _strategy_- Total cost next less costly _strategy_)/ (QALY loss next less costly _strategy_- QALY loss _strategy_); ICER vs. no vaccination = (Total cost _strategy_- Total cost _no intervention_)/(QALY loss _no intervention_- QALY loss _strategy_). Bold ICER: A strategy is cost-effective with ICER < willingness-to-pay threshold (162,401 USD/QALY).
