# Supplementary material for: Cost‐Effectiveness Analysis of Nirsevimab for Respiratory Syncytial Virus Disease Prevention in Newborns of Hong Kong
Source: Influenza Other Respir Viruses. 2025 Oct 1;19(10):e70153. doi: 10.1111/irv.70153 (PMC12485666; doi:10.1111/irv.70153)
Supplement: Supplementary file 10 — Table S6: Scenario 4 (healthcare provider's perspective) results on expected costs and QALY loss per 100,000 infants. [file IRV-19-e70153-s011.docx]

**Supplementary Materials**

**Table S6 Scenario 4 (healthcare provider’s perspective) results on expected costs and QALY loss per 100,000 infants**

| Strategy | Total cost (USD) | QALY loss | ICER vs. next less costly option | ICER vs. no Intervention |
| --- | --- | --- | --- | --- |
| 10% US cost (USD52) |  |  |  |  |
| Nirsevimab catch-up | 8,384,346 | 38.82 | - | **dominant** |
| Nirsevimab year-round | 9,043,924 | 45.71 | dominated | **dominant** |
| Nirsevimab seasonal | 8,335,185 | 60.60 | dominated | **dominant** |
| No intervention | 7,562,816 | 81.52 | dominated | - |
| 25% US cost (USD130) |  |  |  |  |
| No intervention | 7,562,816 | 81.52 | - | - |
| Nirsevimab seasonal | 12,724,473 | 60.60 | dominated | 246,733 |
| Nirsevimab catch-up | 16,179,140 | 38.82 | 201,787 | 201,787 |
| Nirsevimab year-round | 16,840,174 | 45.71 | dominated | 259,072 |
| 50% US cost (USD260) |  |  |  |  |
| No intervention | 7,562,816 | 81.52 | - | - |
| Nirsevimab seasonal | 20,039,955 | 60.60 | dominated | 596,422 |
| Nirsevimab catch-up | 29,170,462 | 38.82 | 506,034 | 506,034 |
| Nirsevimab year-round | 29,833,924 | 45.71 | dominated | 621,924 |

RSV: Respiratory Syncytial Virus; LRTI: lower respiratory tract infections; QALY; quality-adjust life year. ICER: incremental cost per QALY gained; ICER vs. next less costly option= (Total cost _strategy_- Total cost next less costly _strategy_)/ (QALY loss next less costly _strategy_- QALY loss _strategy_); ICER vs. no vaccination = (Total cost _strategy_- Total cost _no intervention_)/(QALY loss _no intervention_- QALY loss _strategy_). Bold ICER: A strategy is cost-effective with ICER < willingness-to-pay threshold (162,401 USD/QALY).
